# Common Household Chemicals and the Allergy Risks in Pre-School Age Children

**DOI:** 10.1371/journal.pone.0013423

**Published:** 2010-10-18

**Authors:** Hyunok Choi, Norbert Schmidbauer, Jan Sundell, Mikael Hasselgren, John Spengler, Carl-Gustaf Bornehag

**Affiliations:** 1 Department of Environmental Health, Harvard School of Public Health, Boston, Massachusetts, United States of America; 2 Norwegian Institute for Air Research, Kjeller, Norway; 3 Department of Building Science, School of Architecture, Tsinghua University, Beijing, China; 4 Primary Care Research Unit, County Council of Varmland, Karlstad, Sweden; 5 Public Health Sciences, Karlstad University, Karlstad, Sweden; 6 SP Technical Research Institute of Sweden, Boras, Sweden; Ludwig-Maximilians-Universität München, Germany

## Abstract

**Background:**

The risk of indoor exposure to volatile organic compounds (VOCs) on allergic airway diseases in children remains unknown.

**Objective:**

We examined the residential concentrations of VOCs, emitted from building materials, paints, furniture, and other lifestyle practices and the risks of multiple allergic diseases as well as the IgE-sensitization in pre-school age children in Sweden.

**Methods:**

In a case-control investigation (198 case children with asthma and allergy and 202 healthy controls), air samples were collected in the room where the child slept. The air samples were analyzed for the levels of eight classes of VOCs.

**Results:**

A natural-log unit of summed propylene glycol and glycol ethers (PGEs) in bedroom air (equal to interquartile range, or 3.43 – 15.65* µ*g/m^3^) was associated with 1.5-fold greater likelihood of being a case (95% CI, 1.1 – 2.1), 1.5-fold greater likelihood of asthma (95% CI, 1.0 – 2.3), 2.8-fold greater likelihood of rhinitis (95% CI, 1.6 – 4.7), and 1.6-fold greater likelihood of eczema (95% CI, 1.1 – 2.3), accounting for gender, secondhand smoke, allergies in both parents, wet cleaning with chemical agents, construction period of the building, limonene, cat and dog allergens, butyl benzyl phthalate (BBzP), and di(2-ethylhexyl)phthalate (DEHP). When the analysis was restricted to the cases, the same unit concentration was associated with 1.8-fold greater likelihood of IgE-sensitization (95% CI, 1.1 – 2.8) compared to the non-IgE sensitized cases. No similar associations were found for the other classes of VOCs.

**Conclusion:**

We propose a novel hypothesis that PGEs in indoor air exacerbate and/or induce the multiple allergic symptoms, asthma, rhinitis and eczema, as well as IgE sensitization respectively.

## Introduction

Global trend in prevalence of allergic airway disease and other types of allergies in children and young adults appears to be stabilizing or even decreasing since the 1990s in “western” countries [1]. However, a substantial portion of the population, especially the children, suffers from these diseases which make it a public health concern. Causal factors underlying these diseases and other contributors of global trend in prevalence since the 1970s remain unknown [2]. This is further highlighted by a growing consensus that genetic predisposing factors do not adequately account for such rapid shift in global prevalence [2]. An emerging body of evidence suggests that environmental conditions during early life are important. In particular, early-life exposure to chemicals commonly found at home, and their possible roles in allergic airway disease, allergic asthma, and rhinitis are speculated [3], [4], [5], [6].

Global secular trend in asthma and the allergy disease prevalence draw a parallel with vast shift in diet, lifestyle, and consumer product uses within the western societies since the World War II [7]. Enormous quantity and array of chemical compounds have been introduced in the societies which adopted western lifestyles [8]. Consumer products, such as computer, TV, and synthetic building materials, including artificial carpets, composite wood, polyvinyl chloride (PVC) flooring, foam cushions, and PVC pipes emit an array of volatile organic compounds (VOCs), semi-volatile organic compounds (sVOCs) and non-organic compounds [8]. VOCs, which predominantly exist in the vapor phase in the atmosphere, and sVOCs, which exist in both vapor and condensed phase, redistribute to indoor surfaces and may persist from several months to years [8]. Both adults and children spend an estimated >90% of daily hours in indoor setting [9]. In addition, energy conservation measures for buildings have led to reduced air exchange rates and promotion of indoor moisture buildup [7], [9].

In infants and children, the role of indoor VOCs as allergens, adjuvants, or mere correlates in development of allergic asthma, and rhinitis remains an open question [6]. Two recent reviews of the literature identified indoor residential chemicals, emitted from particle board, plastic materials, recent painting, home cleaning agents, air freshener, pesticide, and insecticide, consistently increase the risks of multiple allergic symptoms and asthma-like symptoms [10], [11]. However, these studies were limited by small sample sizes, measurement of the complex VOC mixture in terms of the total concentration, and presumption of personal exposure based on the identification of emission related-material or the human activities [11]. Nevertheless, the authors concluded that these epidemiologic studies overall point to a new class of little recognized residential chemical risk factors [11].

In our on-going investigation of the indoor environment and pediatric asthma and allergies, we speculated *a priori* that some constituents of modern organic solvents, which are present, for example, in paints, adhesives, coating agent and cleaning agents as well as other household products/articles, contribute to the asthma and/or allergy diseases. In order to examine complex VOC mixture at low concentration range, we examined the risks of eight classes of VOCs, defined in terms of their chemical properties. Risk of exposure to eight respective VOC classes on multiple allergy diseases and asthma diagnosis was investigated.

## Methods

The present study is part of the on-going Dampness in Buildings and Health (DBH) study which started 2000. At baseline, a modified International Study of Asthma and Allergies in Childhood (ISAAC) questionnaire was given to the parents of all 14,077 children, between the ages 1 – 5, in the county of Värmland, Sweden, cross-sectionally. Responses for 10,851 children were received (response rate, 79%) [12].

One and a half year after the baseline, a follow up questionnaire was sent to the families of 1,056 potential case children (9.7% of the total population). Target population was the children reporting at least two symptoms of wheezing, rhinitis and eczema in both questionnaires. Also, we randomly identified 1,100 symptom free children (representing 48.9% of the population) from local primary care clinics. This resulted in 198 cases and 202 controls.

A medical examination of the 400 children (3–8 years of age) was performed by a team of physicians and nurses during the winter (November, 2001 – March, 2002), simultaneously with home inspections and sample collection.

A case was defined as a child, whose parent reported at least two symptoms of wheezing, rhinitis, or eczema without a cold, during the preceding 12 months on both the baseline and the follow-up questionnaire. Additional outcomes included asthma, rhinitis and eczema. Respective disease status required physician diagnosis in addition to parent-reported persistent symptoms. Diagnostic criteria for current asthma included a combination of (a) three episodes of wheezing prior to age 2; (b) first wheezing episode after the age 2; (c) the first episode of wheezing in a child who also suffers from other atopic diseases; (d) asthma medication use; and (e) an earlier clinical diagnosis of asthma [13]. Lung function test was not performed. Rhinitis case definition required: (a) ever having allergic rhinitis symptoms; (b) symptoms presentation in the nose and/or eyes following the contact with furred animals or pollen; (c) present use of rhinitis medicine. Eczema case definition required at least six months of remitting itching and redness over typical body locations. All children with asthma also met the definition of the case. Among the controls, two children were diagnosed with rhinitis and eight were diagnosed with eczema. Thirteen additional cases were found among the controls. In the present analyses, the 23 misclassified children were excluded from further analysis. The definition of the control status required absence of symptoms without a cold within a year in both rounds of questionnaires. Both cases and controls were excluded if their homes were renovated due to the moisture problems or if they had changed residence since the first questionnaire.

The IgE sensitisation status (yes vs. no) of the child was determined by screening all available children's blood sample (n = 387) for 10 airborne allergens (Phadiatop®, Pharmacia & Upjohn Diagnostics, Uppsala, Sweden); timothy, mugwort, birch, cat, horse, dog, house dust mites (*D. pteronyssinus* and *D. farinae*), and two moulds (*Penicillium*, *Cladosporium*). Cut-off value of 1.2 kUA/l was used to define IgE-positive status.

Informed consent was obtained from all parents. The regional ethics committee at the university hospital in Örebro approved the study.

### Exposure measurements in the homes

Six professional building examiners collected air and dust samples in the room where the child most often slept [14]. Inspectors were blind to the disease status (case vs. control) of the children.

#### VOC sampling

Air samplers were placed 1 meter above the floor in the room. A SKC pocket pump (SKC Pocket Pump 210-1002, SKC Blandford, Dorset, UK) drew in air at 80 ml/min for 60 to 90 minutes (5 to 8 liters) through a Perkin Elmer adsorption tubes (glass, 300 mg Tenax TA). The tubes were sealed with PTFE stoppers, wrapped in alumina foil and kept at −20°C until they were shipped to the Institute for Air Research, Norway for analysis within two weeks of collection. Prior to use the Tenax tubes were cleaned using thermo-desorption at 275°C for 15 minutes for three consecutive cleaning cycles. Use of adsorbent, preparation of adsorbent tubes, sampling equipment, sampling flow and safe-sampling volumes, analytical methods and analytical equipment followed international standards on ambient air quality DIN EN 14662-1 (DIN ISO 5725-2 and 3). There is no standard procedure for VOC sampling and analysis for indoor air, but the chosen method is widely accepted for its reliability [15].

Tenax TA for indoor air analysis is well established with inter-laboratory differences reported to be <10% for benzene analysis (DIN EN 14662-1 annex H2). The temporal stability of compounds trapped on Tenax TA and the formation of artifacts from degradation of the adsorbent Tenax TA is widely discussed in literature [16], [17], [18] – the main degradation products are known as Benzaldehyde, Acetophenone and Benzoic acid and to a minor extent Benzene, Toluene and Xylenes. Other artifacts include aldehydes (Hexanal, Heptanal, Octanal, Nonanal, Decanal), created due to the reactions of ozone from the sample air and fatty acids. All those compounds are also common gases in indoor air. The blank values of those compounds are small compared to the amount of those gases in indoor air with a sample size of more than 5 liter. Due to the chemical structure of Tenax TA (2,6 – diphenylene based polymer), formation of glycol ethers as artifact from Tenax TA is very unlikely and has never been reported.

#### Laboratory Analysis

The VOC samples were analyzed using an automated thermo desorption unit (Perkin Elmer ATD 400, PerkinElmer Inc., Waltham, MA, USA) followed by GC-MS [16], [17], [18]. The samples were desorbed at 250°C - refocused on a Tenax TA cold trap held at minus 30°C and transferred to the GC-MS at 225°C. A Hewlett Packard G 1800 A GC-MS was used as detector maintained at 250°C. The separation column (DB-1701, 32 m length, 1 µm film, 0.32 mm id) was programmed from 40°C to 250°C. The mass range of the detector was from mass 33 to mass 350.

#### Detection and identification

From the Total Ion Chromatogram, the 50 peaks with the largest area were used for further identification routines [16], [17], [18]. An automated mass spectra library check (Wiley) was giving the first preliminary identification results. Each of the library suggestions for component identification were then again cross checked against Norwegian Institute for Airway Research (NILU) 's database for indoor air pollutants which contains about 1,000 components. The database contains retention time indexes from compounds which were identified in indoor air samples at NILU on exact the same analytical system over the last 20 years. Most of the compounds within this database have been verified by direct injection of pure standard solutions or mixed standard solutions. The criteria for identification were over 80% confidence match from the mass spectra library and a match to the retention time database within 5 seconds of relative retention together with a manual check of the retrieved mass spectrogram against the library mass spectrogram. If a peak did not meet those criteria it was named as “unidentified compound”. In every sample the 50 compounds with the highest concentrations were reported as Toluene-equivalents. In addition, the number of all compounds within each sample with a concentration above a baseline-threshold of 0.1 ppb (usually between 180 and 250 compounds) and the concentration-sum of all those compounds were reported [16], [17], [18].

#### Calibration

The calibration was based on toluene equivalents [16], [17], [18]. Ten samples were run together with two standard injections before and after each series. Internal standards consisted of a solution containing 100 ng/µl of benzene, toluene, ethylbenzene and xylenes in methanol [16], [17], [18]. Standard Tenax tubes were prepared by syringe onto the adsorbent followed by 5 minutes of dry nitrogen (20ml).

### Statistical analysis

Detected compounds were grouped into eight classes based on their chemical properties. All eight classes of compounds were natural log (ln) – transformed, considering their right skewed distributions and varying standard deviations (all Komogorov-Smirnov tests >0.05).

We considered whether the quantification of only the 50 most abundant compounds in all homes could have misclassified exposure to PGEs, and might have biased our estimates toward the null. To explore this, we speculated that the variance of the unquantified PGEs would be high, if the variance of the minimum detected concentration of the 405 compounds were high among the 381 samples. The range (0.33 – 11.24* µ*g/m^3^) and the variance (2.31* µ*g/m^3^) of the minimum concentration of the 50^th^ compound were both very low. Overall, the prevalence of the 405 compounds were positively correlated with their concentrations (linear regression coefficient  = 0.73, Figure S1). For example, the variances of limonene (found in 99% of homes) and texanol A (found in 9% of homes) were 211* µ*g/m^3^ and 46* µ*g/m^3^, respectively. Similarly, eight most prevalent PGEs (detected in 165 to 16 homes) were also more likely to have the lowest concentrations range for their minimum value (0.52 – 2.26* µ*g/m^3^), compared to the range of the minimum values (1.42 – 8.29* µ*g/m^3^) for least prevalent PGEs (detected in 1 to 11 homes). Greater likelihood of detecting and quantifying the compounds with low concentration suggest that the unknown PGE concentrations would be very low or absent, supporting the attribution of 1.11* µ*g/m^3^ as a conservative estimate.

The geometric mean concentration of the specific PGEs were compared among the control vs. four outcome groups using Mann-Whitney's U test (*α* = 0.05). In addition, dose-response in effect sizes was examined per quartile increase in bedroom VOC concentrations in air. To further rule out chance finding from multiple comparisons, we examined the association with a clinician diagnosis-free outcome, IgE-sensitization status. While IgE-sensitized controls would have made an ideal reference group, their limited size (n = 23, 11%) did not allow comparison with the sensitized cases (n = 92, 46%). In the resulting case-only analysis, non-sensitized cases were considered a reference group.

The initial model with all potential confounders was reduced through hierarchical backward elimination strategy [19]. In the final model, we controlled for the clinically relevant risk factors regardless of their statistical association (i.e. gender, secondhand smoke exposure at home, allergic disease in both parents), and those factors correlated with both the bedroom concentration and the outcomes. In addition, we adjusted for the phthalate concentration in the bedroom dust, based on our earlier finding of a significant association [20].

## Results

Demographic traits of the cases and controls are shown (Table 1). Other residential characteristics of the children have been described elsewhere [20], [21]. Distribution patterns of the eight classes of VOC are shown (Table 2). The geometric mean and the median concentration of the least common VOCs (i.e. texanols) were about 10-times lower than those for the most prevalent compounds, aromatic hydrocarbons. However, the geometric standard deviations of the most and the least common compounds were very similar, indicating an overall low variability of the concentrations. The number of the detected PGE compounds in given home was highly correlated to the mean concentration (Pearson's coefficient  = 0.58, *p<0.001*). Mean PGE concentration in the homes with 1, 2, 4, and 6 detected compounds were 6.61±8.37, 12.69±9.71, 20.08±8.67, and 71.74±14.41* µ*g/m^3^, respectively.

**Table 1:** Demographic traits of the cases and the controls.

	Control (n = 202)		Case (n = 198)	
BMI [mean ± SD]	16 ± 2		17 ± 2	
Age				
2	1	0%	0	0%
3	41	20%	35	18%
4	32	16%	42	21%
5	44	22%	45	23%
6	36	18%	40	20%
7	45	22%	32	16%
8	3	1%	4	2%
Gender [female]	88	44%	85	43%
ETS exposure at home [yes]	30	15%	40	21%
History of allergic diseases in both parents [yes]	14	7%	50**	25%
Type of building				
Single family house	172	85%	161	81%
Two-family house	11	5%	12	6%
Multifamily house	19	9%	25	13%
Construction period				
Before1960	99	50%	77	41%
1961–1985	68	34%	93	50%
After 1985	31	16%	17	9%
Water leakage during last years [yes]	41	22%	52	29%

**Table 2:** Distribution of the indoor air concentration of VOC classes (µg/m^3^) in 381 homes^a^.

	N	% < functional detection limit b	Geometric mean ± G.S.D.	Minimum	25^th^ pctile	median	75^th^ pctile	Maximum
aromatic hydrocarbons	381	0	41.47±2.53	2.25	21.76	42.76	78.12	458.70
alkanes	381	0	27.89±2.23	3.21	15.33	27.50	48.18	225.70
organic acids	364	4	12.78±1.87	1.23	8.84	13.78	19.92	57.63
aldehydes	330	13	4.71±1.61	0.87	3.53	4.59	6.33	20.69
methyl-alkanes c	288	24	8.68±3.10	0.48	4.22	8.78	17.38	115.85
propylene glycol & glycol ethers^d^	262	31	7.25±2.78	0.46	3.39	7.63	15.60	81.93
dimethyl-alkanes c	124	67	6.10±2.60	0.73	3.08	5.94	11.71	57.99
texanol A+B	95	75	4.33±3.16	0.47	1.83	3.38	11.11	68.69

a) 381 homes due to 9 missing samples and 10 pairs of sibling.

b) Functional detection limit is based on ½ value of the median (1.11 µg/m^3^) of 50^th^ in peak area compound (See Methods).

c) Two samples >5-fold S.D. are eliminated due to likely contamination during air sampling.

d) Includes 1,2-propanediol (CAS # 57-55-6) and 16 glycol ethers shown in Table 3.

### Relationship to asthma and allergy

**Figure 1:**
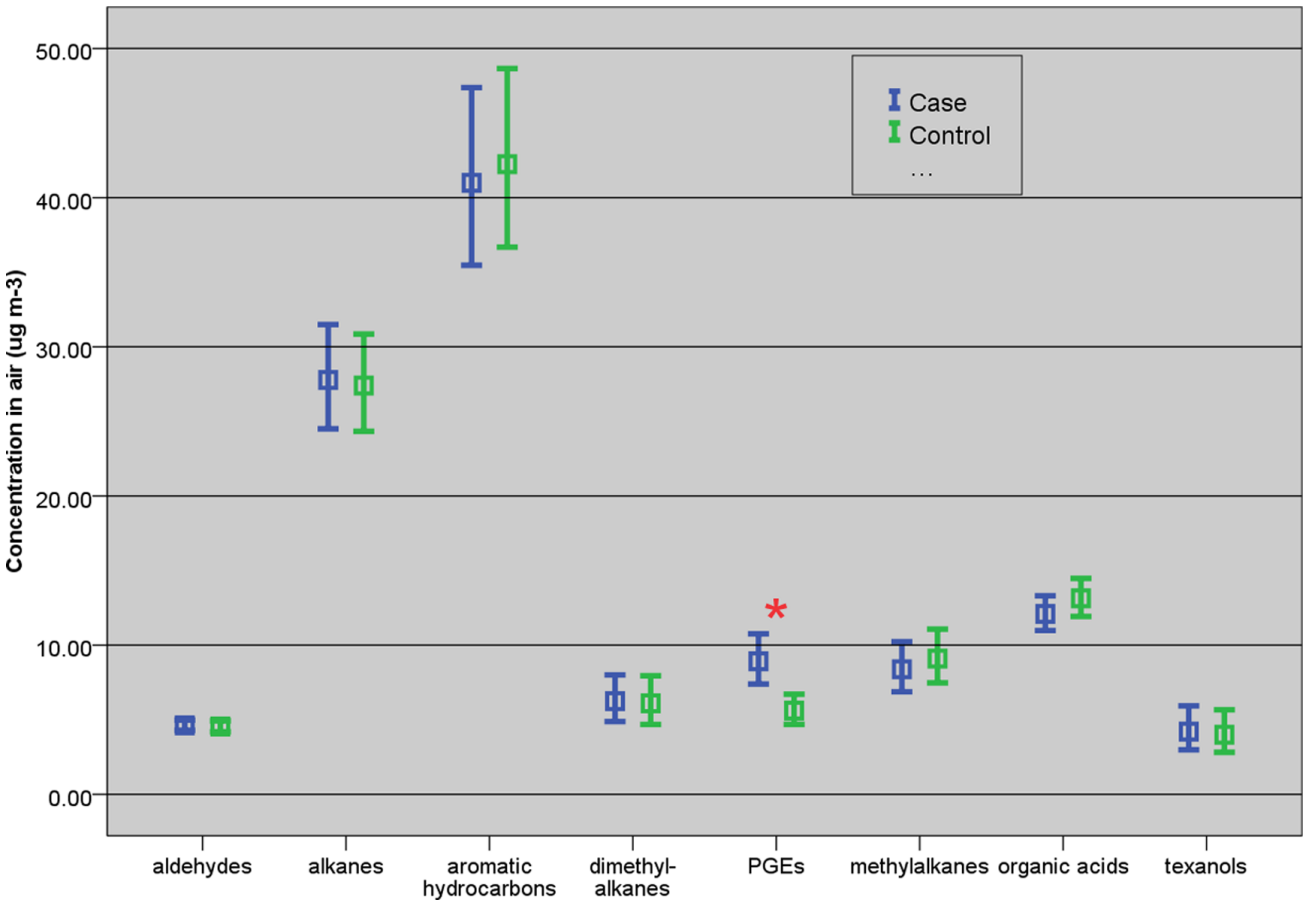
Geometric mean (95% CI) (µg/m^3^) of each VOC classes and compounds for the cases and the controls. PGEs sum of propylene glycol and glycol ethers. * p = 0.001 compared to the controls.

Mean bedroom concentrations of PGEs, but no other classes of VOC, was significantly higher among the cases (9.11* µ*g/m^3^, 95% CI, 7.64 – 10.87* µ*g/m^3^) compared to the controls (5.91* µ*g/m^3^, 95% CI, 4.97 – 7.04* µ*g/m^3^) (*P = 0.001*) (Figure 1). In particular, the geometric mean PGEs for the children with rhinitis (11.29* µ*g/m^3^, 95% CI, 9.09 – 14.02* µ*g/m^3^) were two-fold higher than that in the controls Geometric mean of the individual PGE compounds and Texanol B were consistently higher in the homes of the cases, asthma-, rhinitis- and eczema-diagnosed children, respectively, compared to those in the control homes (Table 3).

**Table 3:** Geometric mean indoor air concentration (µg/m^3^) of glycol, glycol ethers, and texanols in the children's homes.

	Control		Case		Asthma		Rhinitis		Eczema	
	N^a^	geometric mean(95% CI)	N^a^	geometric mean(95% CI)	N^b^	geometric mean(95% CI)	N^b^	geometric mean(95% CI)	N^b^	geometric mean(95% CI)
Propylene glycol & glycol ethers										
1,2-propanediol (propylene glycol)(CAS # 57-55-6)	72	4.55(0.96–21.58)	93	6.43*(1.01–40.89)	57	6.70*(1.14–39.32)	52	6.60*(1.26–34.47)	66	6.50*(0.95–44.76)
1-methoxy-2-propanol(Propylene Glycol Monomethyl Ether)(CAS # 107-98-2)	42	3.49(0.95–12.75)	44	3.81(1.03–14.13)	25	3.29(0.85–12.73)	22	3.90(1.14–13.29)	28	3.59(0.88–14.64)
2-(2-butoxyethoxy)ethanol (CAS # 112-34-5)	31	3.05(0.48–19.25)	38	3.44(0.74–16.07)	26	3.35(0.91–12.33)	22	3.19(1.06–9.62)	27	3.12(0.78–12.44)
1-butoxy-2-propanol (CAS # 5131-66-8)	31	4.51(0.75–27.11)	34	4.38(0.64–30.15)	18	5.23(0.53–51.75)	21	4.92(0.50–48.56)	25	5.21(0.72–37.87)
2-(2-butoxyethoxy)ethanol acetate (CAS # 124-17-4)	13	1.85(0.62–5.50)	20	2.89(0.53–15.72)	12	2.83(0.46–17.37)	14	3.23(0.54–19.26)	11	2.70(0.53–13.81)
2-butoxy ethanol (CAS # 111-76-2)	9	3.00(0.30–29.61)	18	3.11(0.76–12.67)	13	2.81(0.67–11.72)	11	3.40(0.97–11.98)	8	2.68(0.85–8.40)
2-(2-(2-butoxyethoxy)ethoxy) ethanolCAS # 143-22-6)	11	1.55(0.60–3.99)	9	1.85(0.37–9.27)	5	2.60(0.53–12.76)	4	2.45(0.27–21.99)	7	2.45(0.62–9.63)
2-(2-ethoxyethoxy) ethanol (cas # 111-90-0)	7	7.10(1.31–38.47)	9	7.03(1.55–31.96)	7	6.51(1.89–22.45)	5	7.31(1.75–30.62)	5	6.58(1.91–22.67)
1-(2-methoxypropoxy)-2-propanol (CAS # 13429-07-7)	3	3.24(1.85–5.68)	8	5.44(0.80–37.19)	5	5.92(0.81–43.13)	3	15.23(13.02–17.82)	5	5.24(0.52–52.77)
Dipropylene glycol methyl ether (CAS # 34590-94-8)	1	2.55	6	4.19(1.42–12.3)	4	3.88(1.06–14.17)	4	3.55(1.30–9.65)	4	5.31(2.22–12.70)
2-(2-methoxyethoxy) ethanol (CAS # 111-77-3)	3	3.91(2.59–5.90)	3	7.41(2.03–27.04)	3	7.41(2.03–27.04)	2	7.85(1.28–47.95)	2	9.97(3.17–31.36)
2-(2-hydroxypropoxy)-1-propanol (CAS # 106-62-7)	1	0.62	3	1.61(0.76–3.42)	2	1.96(1.21–3.20)	2	1.34(0.76–2.37)	3	1.61(0.76–3.42)
1-(2-methoxy-1-methylethoxy)-2-propanol (CAS # 20324-32-7)		(1.00–1.00)	3	6.33(2.91–13.80)	2	6.13(2.06–18.23)	1	6.76	2	7.84(5.21–11.79)
1-propoxy-2-propanol (CAS # 1569-01-3)	1	1.55	1	8.91	1	8.91	1	8.91		
2-(2-ethoxyethoxy) ethanol acetate (CAS # 112-15-2)			2	8.03(3.99–16.17)	2	8.03(3.99–16.17)	1	10.34	2	8.03(3.99–16.17)
2,2-oxybis ethanol (Diethylene glycol) (CAS # 111-46-6)			1	7.97	1	7.97	1	7.97	1	7.97
Ethanediol (Ethylene glycol) (CAS # 107-21-1)			1	1.92	18	7.28(1.17–45.42)	18	7.82(1.20–51.03)	15	8.11(1.90–34.54)
Texanols										
A	15	8.85(3.74–20.93)	24	7.42(1.38–39.92)	18	7.28(4.71–11.26)	18	7.82(5.01–12.23)	15	8.11(5.55–11.83)
B	39	3.24(0.54–19.45)	49	3.50(0.50–4.56)	36	3.75(2.68–5.24)	27	5.24*(3.52–7.80)	37	3.40(2.50–4.64)

a) Households with compound > functional detection limit.

b) Subset of the case households with compound > functional detection limit.

c) CAS Registry number is unique a identifier for chemical substance (http://www.cas.org/expertise/cascontent/index.html).

*P<0.05 based on Mann-Whitney's U test for each outcome, compared to the control.

A ln-unit increase in PGE concentrations (approximately equal to interquartile range, or 3.43 – 15.65* µ*g/m3) was associated with a significantly greater likelihood of being a case as well as having asthma, rhinitis and eczema diagnosis, respectively, controlling for the gender, secondhand smoke exposure, allergic diseases in both parents, chemical-based cleaning, home construction period (i.e. coded as categorical variable), limonene, ln-transformed cat and dog allergen concentrations, respectively, BBzP, and DEHP concentration in the dust sample (Table 4). In particular, a ln-unit PGEs in air was associated with a 3-times greater likelihood of rhinitis diagnosis (95% CI, 1.6 – 4.7). When the exposure was coded as quartile categories, mean risks of all four outcomes increased dose-responsively, despite that significant risk was observed only for the highest exposure category (≥10.68* µ*g/m3) (Table 4).

**Table 4:** Crude and adjusted odds ratios (95% C.I.) of allergy and asthma diagnoses in children with PGE exposure in indoor air.

		Main exposure variable coding scheme					
	N_case_	one ln-unit(continuous scale)		firstquartile:≤1.11 µg/m^3^	secondquartile:1.12 – 3.45 µg/m^3^	thirdquartile:3.46 – 10.67 µg/m^3^	Fourthquartile:≥10.68 µg/m^3^
		OR (95% CI)		(reference)	OR (95% CI)	OR (95% CI)	OR (95% CI)
Unadjusted a)(N_control_ = 122)							
Case	130	1.5 (1.1 – 1.9)*		1.0	0.9 (0.5–1.7)	1.1 (0.6–1.8)	2.2 (1.3–3.7)*
Asthma	86	1.4 (1.1 – 1.9)*		1.0	1.0 (0.5–2.1)	1.0 (0.6–1.9)	2.3 (1.2–4.1)*
Rhinitis	71	2.1 (1.5 – 2.9)*		1.0	0.5 (0.2–1.2)	1.3 (0.7–2.5)	2.7 (1.4–5.0)*
Eczema	92	1.6 (1.2 – 2.2)*		1.0	1.0 (0.5–2.0)	1.0 (0.6–1.9)	2.6 (1.4–4.6)*
Adjusted a, b)(N_control_ = 167)							
Case	155	1.5 (1.1 – 2.1)*		1.0	0.8 (0.3–1.8)	1.4 (0.7–2.8)	2.3 (1.2 – 4.7)*
Asthma	93	1.5 (1.0 – 2.3)*		1.0	0.8 (0.3–1.9)	1.3 (0.6–2.7)	2.0 (0.9 – 4.4)
Rhinitis	74	2.8 (1.6 – 4.7)*		1.0	0.5 (0.1–1.8)	2.4 (1.0–5.8)	4.2 (1.7 – 10.3)*
Eczema	100	1.6 (1.1 – 2.3)*		1.0	0.9 (0.4–2.2)	1.2 (0.6–2.7)	2.5 (1.1 – 5.3)*
sum of 16 glycol ethers, excluding propylene glycol a, b)(N_control_ = 162)							
Case	153	1.3 (1.0–1.6)*					
Asthma	101	1.2 (1.0–1.6)*					
Rhinitis	74	1.7 (1.3–2.4)*					
Eczema	108	1.3 (1.0–1.7)*					
Cases only a,c)(IgE-negative = 54)							
IgE-positive	53	1.8 (1.1 – 2.8)*		1.0	0.5 (0.1–1.9)	1.6 (0.6–4.3)	2.2 (0.9 – 5.8)

a) Subjects < functional detection limit of glycol ethers were attributed one half-value (1.11 µg/m^3^).

b) Accounts for gender, secondhand smoke exposure, allergic diseases in both parents, chemical-based cleaning, home construction period (i.e. coded as categorical variable), limonene, ln-transformed cat and dog allergen concentrations, respectively, BBzP, and DEHP concentration in the dust sample. Subject size is reduced because only dust samples >25 mg are considered [20].

c) Analysis was restricted to the cases only and the model accounted for same variables as the adjusted model.

*P –value <0.05

**Figure 2:**
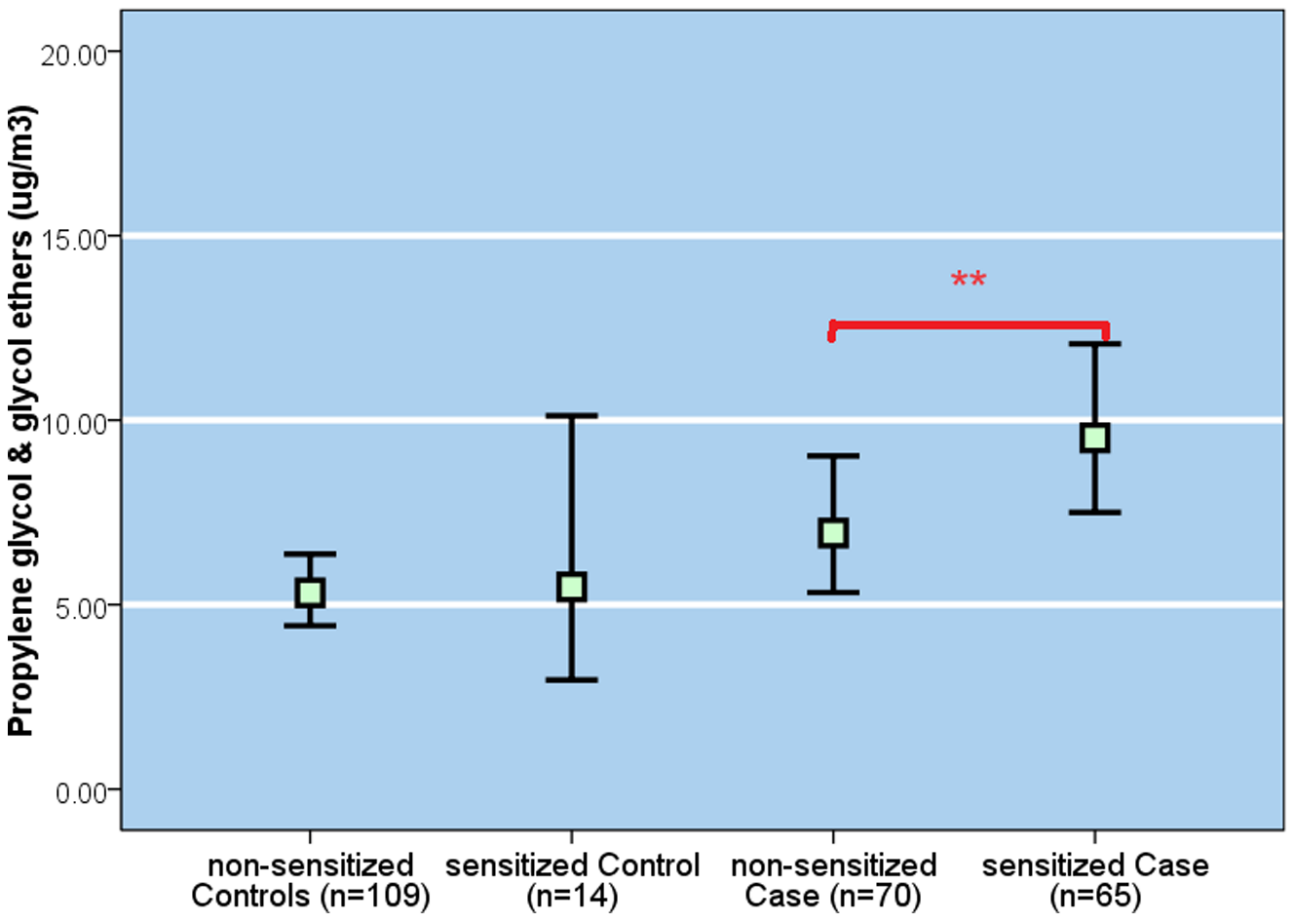
Geometric mean (95% CI) (µg/m^3^) of propylene glycol & glycol ethers in the bedroom air. ** p<0.001 compared to non sensitized cases tested based on T-test.

**Figure 3:**
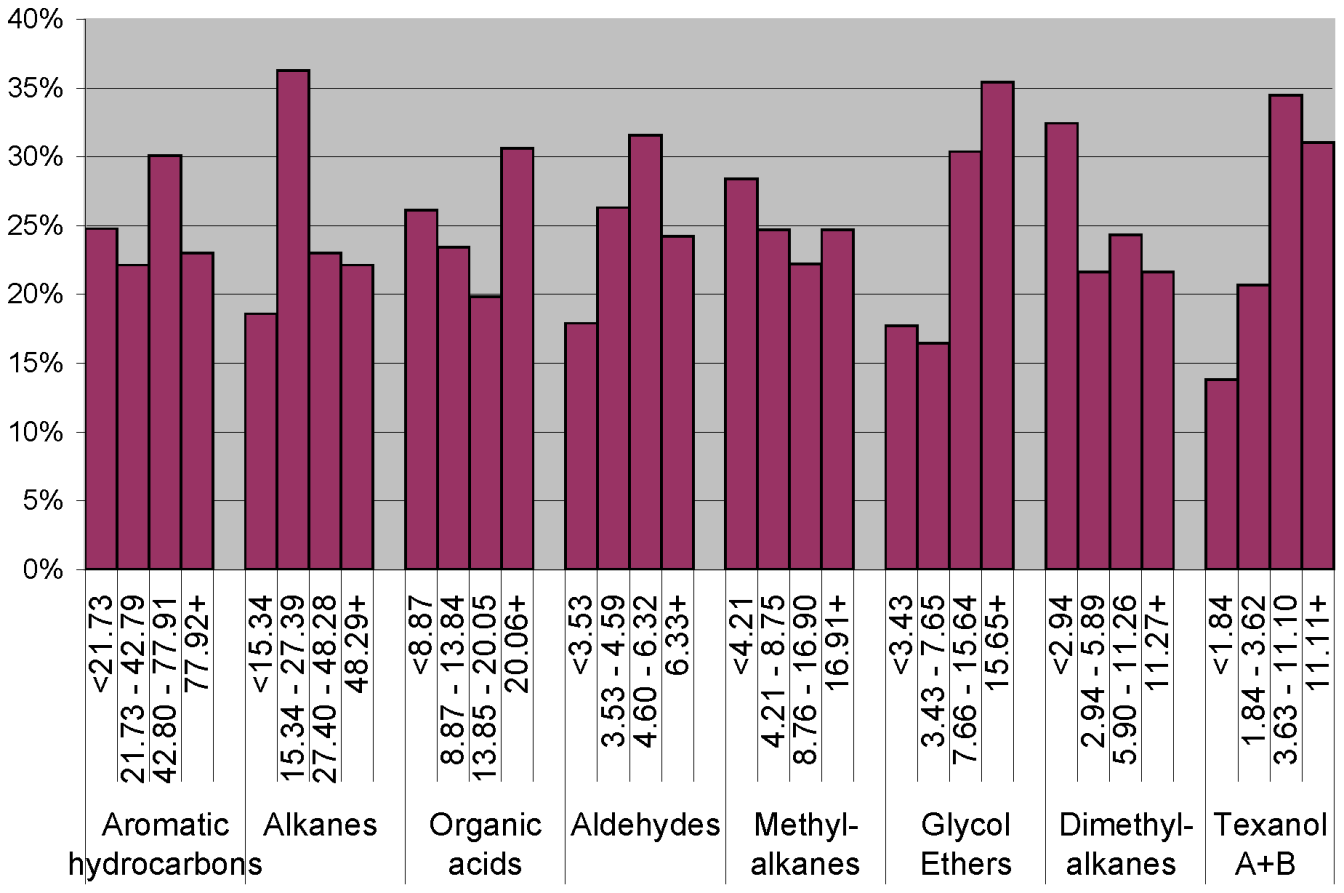
Prevalence of IgE-positive among the cases.

Geometric mean PGE concentration of the IgE-positive cases was significantly higher than that for the IgE-negative cases (*P<0.001*, Figure 2). Also, the prevalence of IgE-positive case status increased in dose-responsive manner per quartile PGE exposure unit (Figure 3) in the sample restricted to the cases only. There was no relationship between IgE-sensitization and other compound classes. In the model adjusting for the same set of confounders as above, a ln-unit PGE concentration was associated with a significant, two-fold increase in the likelihood of IgE-sensitization whether the PGEs were coded in a continuous scale (95% CI, 1.2 – 3.0), or comparing highest to the lowest quartile categories (95% CI, 0.9 – 5.2) (see Table 4). In the analysis restricted to the eczema-diagnosed children with similar level of skin lotion use, geometric mean PGEs were significantly higher among sensitized eczema-diagnosed children compared to non-sensitized children (3.59 vs. 2.47* µ*g/m^3^, *p* = *0.020*).

Finally, Texanol B level was significantly higher among the IgE-sensitized cases (n = 24; GM ± GSD, 5.61±2.78* µ*g/m^3^) compared to the non-sensitized cases (n = 24; 2.23±2.78* µ*g/m^3^; *p = 0.001*). But, Texanol A was not found in sufficient number of homes to allow a meaningful comparison (n = 27).

Considering that propylene glycol was the most common PGEs, we also examined whether the apparent risks were driven by this compound. A ln-unit propylene glycol concentration was associated with a significantly elevated likelihood of the case status (aOR  = 1.5, 95% CI, 1.0–2.3), asthma (aOR  = 2.1, 95% CI, 1.2–3.6), rhinitis (aOR  = 2.4, 95% CI, 1.2–4.6), but, not eczema (aOR  = 1.4, 95% CI, 0.8–2.3) compared to the control children, controlling for the same set of confounders as before. When the main exposure variable was alternatively defined as the sum of 16 glycol ethers, excluding propylene glycol, the likelihood of the four respective outcomes were essentially very similar to those posed by sum of 17 glycol ethers including propylene glycol (Table 4). When restricted to the cases only, a ln-unit exposure of the summed 16 glycol ether compounds was associated with a 1.4-fold greater likelihood of IgE-sensitization (95% CI, 1.0 – 1.90).

### Potential confounders

Self-reported cleaning ≥ once/week and history of allergic diseases in both parents remained significant predictors of all outcomes in the final multivariate model. As the mean PGE concentration was higher for those who wet-clean frequently, and has also been shown to pose an independent risk, wet-cleaning could have confounded the apparent associations. To examine this, same logistic regression analysis was conducted by stratifying the data according to the wet cleaning frequency. The adjusted likelihoods of all outcome were not different by >30% for the frequent cleaners (≥ once/week), compared to the reference group (clean < once/month), or the overall aORs. Furthermore, limonene, a marker compound of the chemical-based cleaning agent, was neither an independent risk factor, nor a confounder in the final model.

We have earlier reported that the concentration of di(2-ethylhexyl)phthalate (DEHP) and n-butyl benzyl phthalate (BBzP), respectively, in the indoor dust significantly increase the risks of allergic symptoms in children from the same data set [20]. In the present analyses, the phthalate concentration did not affect the estimated PGE risks, suggesting that they are unlikely to have confounded the associations. BBzP remained an independent risk factor of the rhinitis (aOR = 1.8; 95% CI, 1.1 – 3.0) after accounting for the effect of the PGEs. Also, in the adjusted model, the risk of DEHP on asthma remained considerably high (aOR  = 1.7 for highest quartile category; 95% CI, 0.7 – 4.0).

## Discussion

Goal of the present analysis was to investigate the independent risks of each class of VOCs at home, controlling for the known and potential demographic correlates of both the bedroom VOC concentrations and the outcomes. In the present group of children, only PGEs in indoor air significantly increased the risks of multiple allergic symptoms/diseases (i.e. case status), asthma, rhinitis and eczema, respectively. In addition, a ln-unit PGE concentrations also increased the likelihood of IgE-sensitization among the cases while similar associations were not found for the other VOC classes. At the same time, roles of Texanol A and B require further examination due to their moderate correlation with the PGE levels, and demonstrated risks on asthma symptoms in prior investigations [22].

Several lines of evidence support that our findings are not due to a chance or a bias. First, concentration-dependent increases in the likelihood for all outcomes were observed per ln-unit concentration, or per quartile categories.

Second, apparently elevated likelihood of the present outcomes was not driven by propylene glycol, most abundant PGE compound. Rather, the estimated risks after excluding the propylene glycol were almost identical as those based on the original definition of the PGEs. Furthermore, mean risks of the number of the PGE compounds in given home posed comparable risks as those estimates using the PGE concentrations, shown in Table 4. This suggests that multiple compounds, rather than a single one, contribute to the observed risks.

Third, water-based cleaning does not confound the PGE-outcome associations, and, thus, does not raise potential for a reverse causality. This is supported by similarity in risks across the families with varying cleaning frequencies. In addition, limonene was neither an independent risk factor (aOR  = 1.15; 95% CI, 0.86 – 1.53) of the case status, nor a confounder of PGE-asthma/allergy associations. Similarly, the terpene hydrocarbons did not pose an independent risk on any of the outcomes, or confound the PGE-asthma/allergy associations. Furthermore, among the cases, a unit PGE exposure increased the likelihood of IgE-sensitization by 1.3- and 1.7-fold within the families that clean ≥ once/wk and every other week, respectively. Such independent validation of PGEs-IgE sensitization suggests that our diagnosis dependent outcomes are not merely correlated with frequent cleaning.

Fourth, a history of repainting at least one room in the house pre- or post-natal to the birth of the child was associated with a 63% increase in mean PGEs compared to those who never repainted in a group of families with higher than the median excess moisture level (≥2.158 g/m^3^, *p for interaction  = 0.03*). Thus, repainting might have provided a sustained exposure since the gestational period or shortly following the birth. This is because the information regarding the history of repainting was collected 1.5 year prior to the onset of the case-control study. Since the initial cross-sectional investigation, all parents reported that they remained in the same house and have not changed most life-style practices. To further validate this, the families that renovated their house due to flooding were excluded. This suggests that the temporal window of exposure occurred prior to the initial symptom presentation while the present cohort was critically vulnerable to these chemical compounds.

Multiple indoor sources emit PGEs. A growing body of literature suggest either the PGE sources (self-reported by the subjects), or their directly quantified concentrations exacerbate airway problems. Propylene glycol and glycol ethers are a diverse group of compounds with superior solvent and coalescent properties [23]. Due to their lower volatility and higher degree of solvency, they are widely used in water-based paint, varnishes, cleaning fluids, pharmaceuticals, pesticides, cosmetics, and processed foods [24]. For non-solvent purposes, they are used in PVC pipes [22], hydraulic and brake fluids, de-icing fluids for aircrafts, and artificial theatrical smoke [25]. 1-methoxy-2-propanol was the most prevalent glycol ether compound in the present investigation. In a group of healthy adult volunteers, administration of 309 mg/m^3^ of propylene glycol, and 35 mg/m^3^ of glycol ethers and texanol mixture, respectively induced acute eye, nose, throat irritation and dyspnea [26], [27]. However, our observed PGE concentration range (i.e. <82* µ*g/m^3^) is more than 400-folds lower than the exposure ranges reported in occupational and experimental settings [26], [27]. In a prospective cohort study of house-painters from 1989 – 1991, occupational exposure to water-based paint led to a significantly higher incidence of chest tightness/wheezing, airway irritation, bronchial hyper-responsiveness, and shortness of breath [23]. In a non-occupational setting, greater likelihood of asthma symptoms have been observed in adults exposed to a newly painted wood or kitchen surfaces [28], and/or synthetic material-based furniture [5], [29]. Finally, in the Avon cohort study, a household chemical exposure score was associated with late onset wheezing as well as reduced lung function in children 8.5 years of age [10].

While the mechanism through which glycols and glycol ethers affect the allergic responses is not well understood, it has been known for more than three decades that inhalation of vaporized propylene glycol methyl ether (PGME) induce mucous membrane irritation in nasal passage way of humans [30] and rats [31]. Administration of human nasal respiratory epithelial cell line from healthy individuals with 1-methoxy-2-propanol *in vitro* acutely induced the transcription of TNF-α, IL-1β, and IL-6, markers of early inflammatory response, as well as Cyclooxygenase 2 (COX-2) for the following 4-hours [32]. However, sustained response was observed only for COX-2, a key enzyme in the prostaglandin synthesis during inflammatory responses [32]. Overall, the question of long-term airway injury from the glycol ethers and other organic solvent exposure requires clarification [33].

### Glycol ethers as endocrine disrupting chemicals

Several glycol ether compounds join a growing list of sVOCs that are suggested to contribute to allergic diseases in humans [4]. While several PGEs are well-known endocrine disruptors, very little is known whether and how they influence developing immune system. For example, 1-methoxy-2-propanol, a beta-isomer of PGME, was the most common glycol ethers in the homes of our cohort. Administration of 1-methoxy-2-propanol, during gametogenesis phase of the parent rats could induce >50% reduction of testicular and epididymal sperm counts [34]. Furthermore, 1-methoxy-2-propanol exposure in the parental generation of rats significantly alter the sex ratio, the fetal loss, and birthweight reduction in two subsequent generations [34]. In addition to these effects, our results suggest the PGEs may pose wider range of health effects than previously known. Considering low range of exposure in the present observation, experimental model should be developed not only to validate our results, but also to clarify the mechanism of PGE effects in developing airways and reproductive systems.

### Limitations of the study

We examined whether the limitations in sampling and laboratory analysis misclassified the exposure scenarios for the children, and resulted in a biased estimates of the risks.

First, the airborne VOC levels were measured on the prevalent cases and the controls. VOC exposure characteristics during the period of the child's critical developmental window and temporal stability of the exposure traits to the present study period (at age 3 – 8) remain unknown. While the temporal reliability of the VOC concentrations could not be directly ascertained here, the parental responses from the two rounds of questionnaires suggest that the home indoor environment has not changed between the baseline and the present investigation. We also excluded families that renovated or moved their residence. In addition, while the likelihood of the outcome diagnosis as well as the severity is often positively correlated with the age of the child, we could not examine whether the age modifies the PGE risks due to the limited sample size.

Second, the possibility that the cases and the controls differ in the likelihood or in the concentration range of the missing PGE concentrations was examined. Under this scenario, the detected PGE concentration range would systematically differ between the cases and the controls, resulting in biased estimates of the risks away from the null. Proportion of the cases and controls with unknown PGE concentrations were comparable (28% vs. 37%). The interquartile ranges of the minimum of the 405 compounds were almost identical between the cases and the controls (Figure S2). This suggests that our sampling methods and laboratory analysis are unlikely to have artificially inflated the true concentrations among the cases only. In a subset of children whose sensitization status was determined independent of the clinical diagnoses, the mean PGE levels were significantly higher for the sensitized children, compared to either the non-sensitized children. Similar association was absent for all other VOC classes. As a result, systematic difference in concentration ranges of 50 compounds between the cases and the controls were ruled out.

Third, we did not measure the frequency and extensiveness of the skin lotion use on the child as possible source for PGEs. This might have also contributed to an exposure misclassification. Transdermal exposure to PGEs has been suggested an important exposure route [35], [36]. In a group of 28 healthy male printing workers chronically exposed to 2-(2-butoxyethoxy)ethanol, urinary level of its chief metabolite, 2-(2-butoxyethoxy)ethanol butoxyethoxyacetic acid, was elevated particularly among those with skin lesion [35]. Evidence of transdermal absorption was observed in all workers with erythema and scaliness of skin [35]. While the children could have been exposed to PGE through skin lotion application in this study, this does not account for the present association.

Fourth, due to high mutual correlations of the PGE compounds, and their overall low concentrations, we currently cannot distinguish the risks of the individual compounds.

### Internal Validity of VOC Sampling and the Laboratory Analysis

Our VOC sampling approach with adsorption/thermal desorption coupled with gas chromatography-mass spectrometry (GC/MS) has been validated as sensitive, simple, and cost-effective assessment method [15], [37], [38]. Also, our sampling duration (60–90 min) was substantially longer than the standard protocol [39]. Compared to other methods, our present approach has an advantage of higher sensitivity [39]. In a number of indoor VOC investigations, which relied on Tenax TA as a general purpose adsorbent, overall very low inherent artifacts were observed [16]. Known artifacts of Tenax TA do not include glycol ethers. Thus, glycol ethers are unlikely to have been introduced in this investigation as sampling artifacts [39]. At the same time, no study as ever examined temporal stability of the 405 compounds in this study over time. A time period of 5 to 6 weeks between sampling and analysis could influence the artifact level and recovery rates. While this is likely to have biased the aromatic hydrocarbon level towards the null, there was no evidence that the PGEs were also influenced by the transport duration. The method used is in accordance to best laboratory practice and the recommendations given by DIN EN 14662-1 and Helmig 1996 [16].

The prevalence and concentration the VOCs detected in the present study are strikingly concordant with those detected from other Scandinavian countries. We compared the concordance of our detected VOCs with Finnish EXPOLIS study [40]. In the EXPOLIS study, air samples (2–3 L) from 183 homes were collected during the winter of 1996–1997 in Helsinki, Finland, focusing on 30 VOCs as target compounds. Extensive quality assurance and control standards were practiced. Of the 30 VOCs, 21 VOCs were also collected in our study. The prevalence (% detected in participant homes) of the VOC compounds were significantly correlated between the two studies (R^2^  = 0.57, *p<0.001*) (Figure S3). Also, eight VOC compounds, which were identified in ≥80% of the homes in both the studies (i.e., toluene, limonene, hexanal, p/m-xylene, benzaldehyde, octanal, undecane, and ethylbenzene), their concentrations were significantly correlated (R^2^ = 0.612, *p = 0.022*) (Figure S4). This suggests that compounds with low prevalence are also expected to have low concentrations in both DBH and EXPOLIS. For example, 2-methyl-1-propanol, observed in 5% of the homes of DBH study was 1.96* µ*g/m^3^ and 3.37* µ*g/m^3^ in EXPOLIS. Striking similarities in absolute concentration and correlation of the compounds between the two studies support the validity of our sampling and analytical procedures.

### Summary

Here, the present investigation demonstrates for the first time that the bedroom concentration of PGEs are significantly associated with an elevated risks of multiple allergic symptoms, rhinitis and eczema, respectively, as well as IgE-sensitization in preschool age children. Apparent risks of PGEs at such low concentrations at home raise concerns for the vulnerability of infants and young children. Our present observations warrant confirmation in a prospective cohort study. Clarification of the underlying mechanism of the PGE effects on developing human immune system is also necessary.

## Supporting Information

Figure S1 Relationship between prevalence of each VOC compound and concentration variance.
(0.01 MB PDF)

Figure S2 Distribution of the minimum detected concentration in the cases and the controls (n = 381).
(0.03 MB PDF)

Figure S3 Comparison of the percentage of homes where VOCs were detected in the DBH and the EXPOLIS study. (Pearson correlation R2 = 0.568, p<0.001).
(0.03 MB PDF)

Figure S4 Comparison of indoor air geometric mean concentrations for VOCs found in at least 80% (see Figure 2) in the homes in both the DBH and the EXPOLIS study. (Pearson correlation R2  = 0.612, p = 0.022.)
(0.03 MB PDF)
