# Correction: Common Household Chemicals and the Allergy Risks in Pre-School Age Children

**DOI:** 10.1371/annotation/d452fac2-f6e6-4aec-88f6-51535b27c9da

**Published:** 2011-06-06

**Authors:** Hyunok Choi, Norbert Schmidbauer, Jan Sundell, Mikael Hasselgren, John Spengler, Carl-Gustaf Bornehag

The second sentence under the subhead "Glycol ethers as endocrine disrupting chemicals" in the Discussion section should be: "For example, 1-methoxy-2-propanol, an alfa-isomer of PGME, was the most common glycol ethers in the homes of our cohort"

