# Correction: Common Household Chemicals and the Allergy Risks in Pre-School Age Children

**DOI:** 10.1371/annotation/4288210e-9577-4510-9374-6c16037a2224

**Published:** 2011-06-06

**Authors:** Hyunok Choi, Norbert Schmidbauer, Jan Sundell, Mikael Hasselgren, John Spengler, Carl-Gustaf Bornehag

The use of the terms "Texanol" and "Texanol A" and "Texanol B" in the article can be misleading and should therefore be replaced by the two compounds' formal name and Cas-number. Texanol A should be replaced by Propanoic acid,2-methyl-, 1-(2-hydroxy-1-methylethyl)-2,2-dimethylpropyl ester (Cas-number 74367-33-2) and Texanol B should be replaced by Propanoic acid,2-methyl-, 3-hydroxy-2,4,4-trimethylpentyl ester (Cas-number 74367-34-3).

