# Supplementary figures and images for: Common Household Chemicals and the Allergy Risks in Pre-School Age Children
Source: PLoS One. 2010 Oct 18;5(10):e13423. doi: 10.1371/journal.pone.0013423 (PMC2956675; doi:10.1371/journal.pone.0013423)

Figure S1. Relationship between prevalence of each VOC compounds and concentration variance.

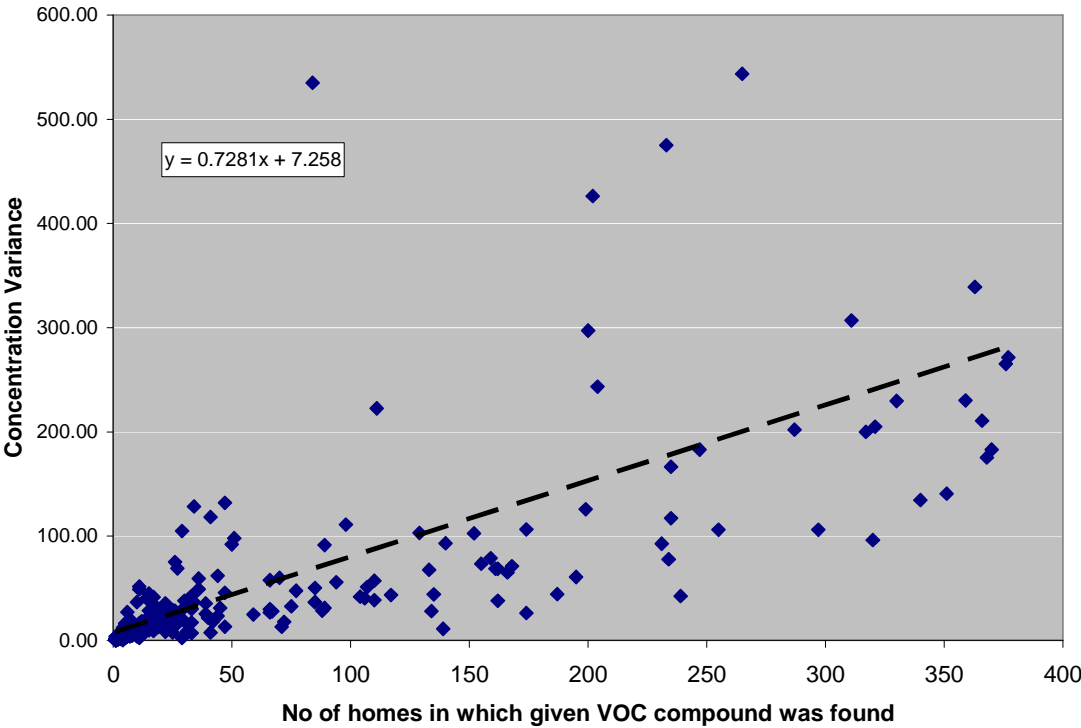

Supplement: Figure S1 — Relationship between prevalence of each VOC compound and concentration variance. (0.01 MB PDF) [file pone.0013423.s001.pdf]

Figure S2. Distribution of the minimum detected concentration in the cases and the controls (n = 381).

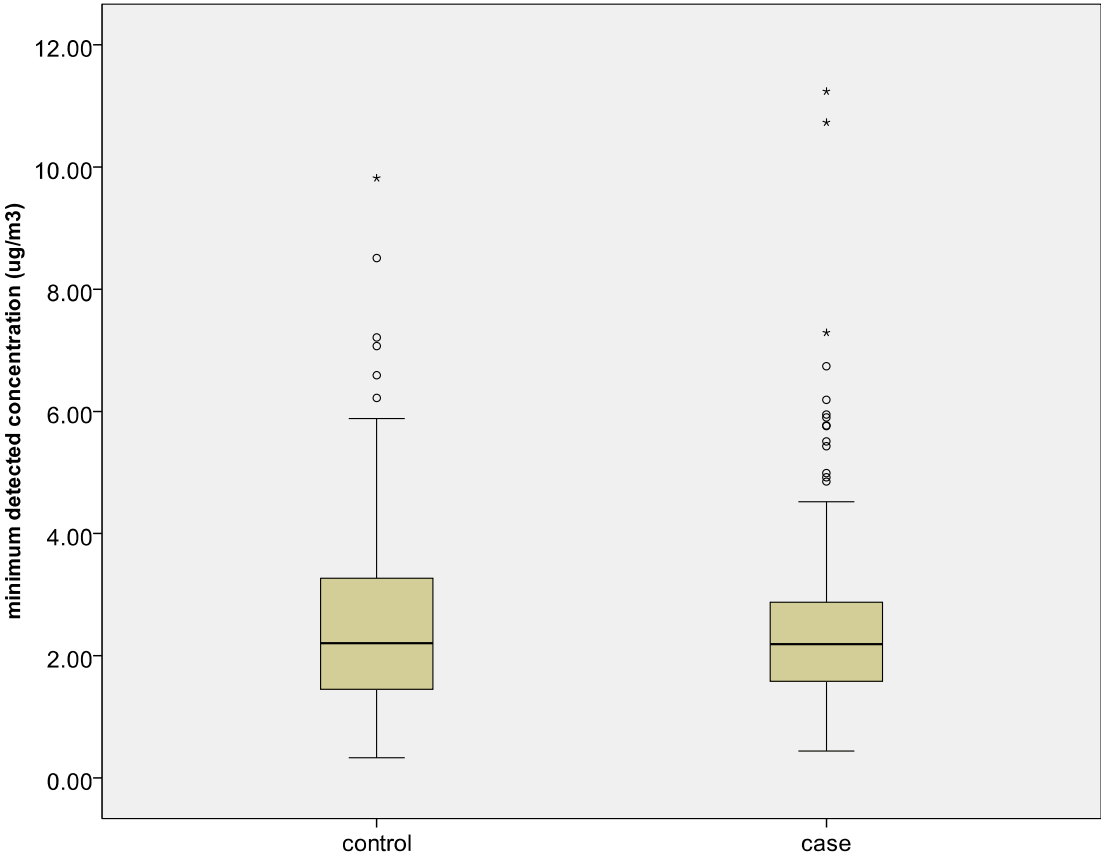

Supplement: Figure S2 — Distribution of the minimum detected concentration in the cases and the controls (n = 381). (0.03 MB PDF) [file pone.0013423.s002.pdf]
