# Supplementary material for: Common Household Chemicals and the Allergy Risks in Pre-School Age Children
Source: PLoS One. 2010 Oct 18;5(10):e13423. doi: 10.1371/journal.pone.0013423 (PMC2956675; doi:10.1371/journal.pone.0013423)

Figure S3. Comparison of the percentage of homes where VOCs were detected in the DBH and the EXPOLIS study. (Pearson correlation R^2=0.568, $p<0.001$).

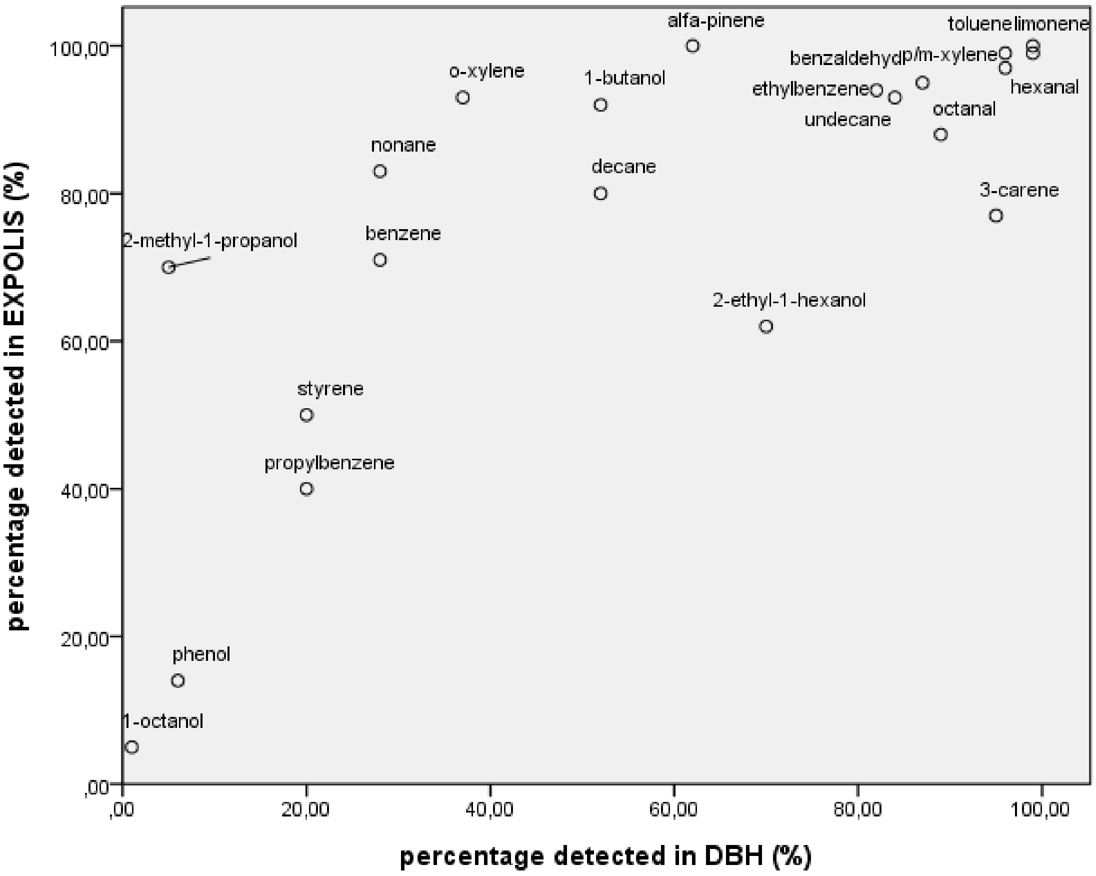

Supplement: Figure S3 — Comparison of the percentage of homes where VOCs were detected in the DBH and the EXPOLIS study. (Pearson correlation R2 = 0.568, p<0.001). (0.03 MB PDF) [file pone.0013423.s003.pdf]
