# Supplementary material for: Common Household Chemicals and the Allergy Risks in Pre-School Age Children
Source: PLoS One. 2010 Oct 18;5(10):e13423. doi: 10.1371/journal.pone.0013423 (PMC2956675; doi:10.1371/journal.pone.0013423)

Figure S4. Comparison of indoor air geometric mean concentrations for VOCs found in at least 80% (see Figure 2) in the homes in both the DBH and the EXPOLIS study. (Pearson correlation R^2 =0.612, p=0.022).

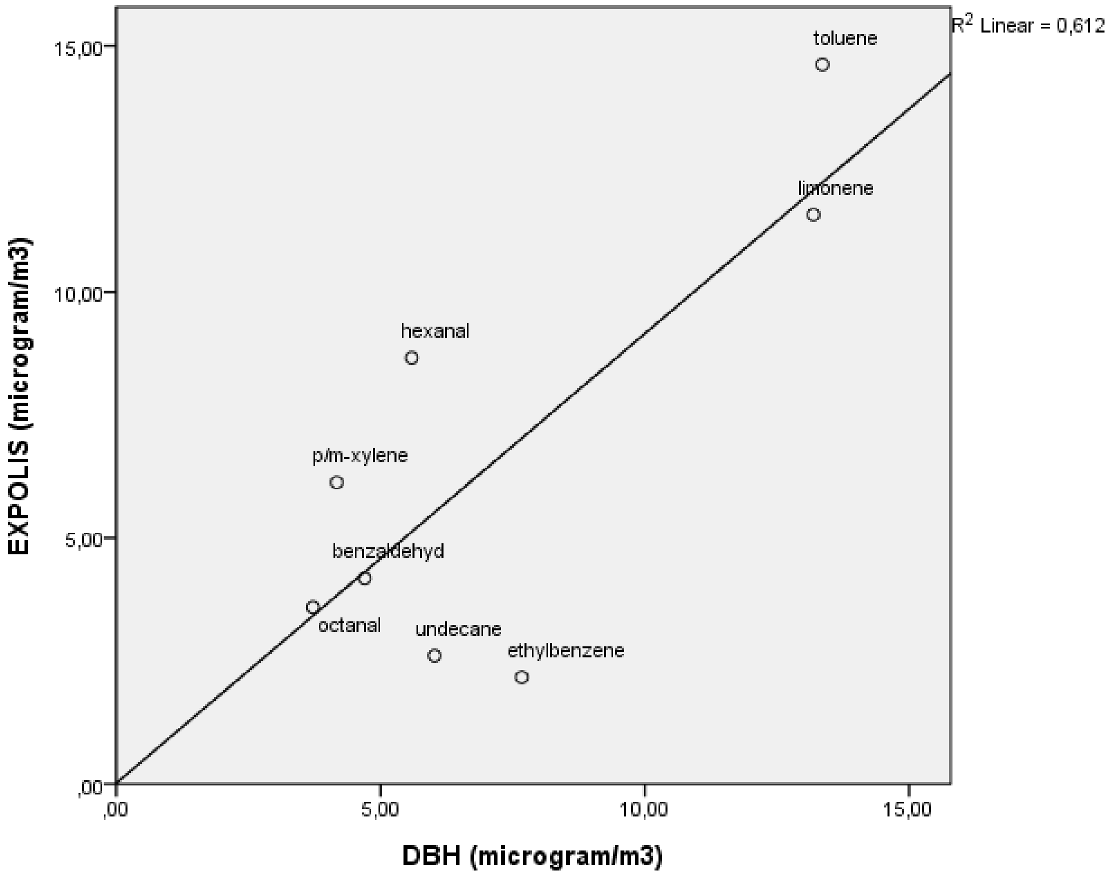

Supplement: Figure S4 — Comparison of indoor air geometric mean concentrations for VOCs found in at least 80% (see Figure 2) in the homes in both the DBH and the EXPOLIS study. (Pearson correlation R2 = 0.612, p = 0.022.) (0.03 MB PDF) [file pone.0013423.s004.pdf]
